# Acupuncture therapy for chronic low back pain: protocol of a prospective, multi-center, registry study

**DOI:** 10.1186/s12891-019-2894-4

**Published:** 2019-10-27

**Authors:** Xuqiang Wei, Baoyan Liu, Liyun He, Xingyue Yang, Jincao Zhou, Hong Zhao, Jia Liu

**Affiliations:** 1 College of Acupuncture and Orthopedic, Hubei University of Chinese Medicine, No.1 Huangjiahu West Road, Hongshan District, Wuhan, Hubei 430065 People’s Republic of China; 2 Institute of Basic Research in Clinical Medicine, China Academy of Chinese Medical Sciences, No.16 Dongzhimen inside south St. Dongchen District, Beijing, 100700 People’s Republic of China; 3 School of Acupuncture-Moxibustion and Tuina, Beijing University of Chinese Medicine, No. 11, North Third Ring Road, Chaoyang District, Beijing, 100045 People’s Republic of China; 4 Institute of Acupuncture and Moxibustion, China Academy of Chinese Medical Sciences, No.16 Dongzhimen inside south St. Dongchen District, Beijing, 100700 People’s Republic of China

**Keywords:** Acupuncture, Registry, Chronic low back pain, Real-world, Protocol

## Abstract

**Objective:**

Acupuncture therapy for chronic low back pain (CLBP) has received increasing attention. Nevertheless, the evidence of efficacy and safety of random controlled trials (RCTs) remains controversial. Acupuncture as a complex intervention influenced by many factors, its effectiveness in treating chronic low back pain in the real world is unknown. We will develop a network-based registry study to evaluate the effectiveness and safety of acupuncture for the treatment of chronic low back pain and explore key factors affecting efficacy in the real world.

**Methods:**

A prospective, multi-center and dynamic registry study. All acupuncture related information will be collected through a high-quality structured network platform. Patients with CLBP included in the study met the following criteria: age from 16 to 80 years, using acupuncture as a main therapy and voluntarily signing the informed consent. At least 2000 patients, 27 acupuncturist, and 9 medical centers will be recruited under actual clinical settings at the first stage. Numeric rating scale (NRS), Oswestry Disability Index (ODI) and Effective rate will be measured in pain and functional disability assessment, respectively, as the primary outcome. Evaluation index will be collected at the baseline and follow-up in 1, 4, 12 weeks after the last visit. Hierarchical models and regression analysis will be used to explore the key factors affecting acupuncture effectiveness. Effects between propensity matching groups (Traditional Chinese acupuncture style vs Microacupuncture style, Local acupoint selection vs Non-local acupoint selection, Single Acupuncture vs Combined therapy) will be compared.

**Discussion:**

This study will be conducted based on the characteristics of acupuncture therapy in the “Real World”. Fundamental factors affecting the clinical effectiveness of acupuncture and the preferred acupuncture regimen in the treatment of CLBP will be identified. Reliable acupuncture evidence for the treatment of CLBP through the registry will be a significant supplement to the RCTs.

**Trial registration:**

Chinese Clinical Trial Registry, ChiCTR-OOC-17010751 and Acupuncture-Moxibustion Clinical Trial Registry, AMCTR-OOO-17000045. Registered date on 3 December 2016.

## Background

As a consequence of the acceleration of global aging, sedentary lifestyle increasing and average weight gain, chronic low back pain (CLBP) has become a global condition with high incidence. Although disability and financial burden attributed to chronic low back pain is substantially different between countries, the incremental impact of the worldwide health care system predicted to tremendously in the coming decades [1]. According to 2015 global statistics, low back pain accounts for 7.3% point prevalence of activity-limiting illnesses, affected 5.4 billion people at all ages [2]. CLBP accounts for a significant portion of the $100 billion spent annually on treatment in the United States [3]. In China, back pain kept the top one disorder that causes of years lived with disability(YLDs) from 1990 to 2010 [4]. Therefore, effective and safe treatment is especially crucial in overcoming low back pain and disability related to the chronic condition.

In a variety of treatment options devoted to back-related disability and chronic pain consequence, non-invasive treatment therapies captured more attention. Changes can be verified in recent guidelines for low back pain [5–7]. Interventional procedure and operation are limited to recommend in guidelines, as well as the pharmacotherapy, in consideration of efficacity, safety and accessibility. Moreover, nonpharmacological intervention was recommended as first-line treatment, which included acupuncture, massage, spinal manipulation, and yoga [6]. As a characteristic non-pharmaceutical option against the opioid crisis, acupuncture captured particularly concerning in pain relief. On account of safety and the actual effect, acupuncture is widely accepted in the management of chronic low back pain [8, 9]. Low back pain was the top 1 frequently treated indication in US’s acupuncture clinic according to a cross-sectional study [10]. However, there is a lack of basic dataset on the use of acupuncture to treat CLBP in China and abroad, including the effective population characteristics, reasonable acupuncture session, preferred acupuncture protocol and acupuncture implementation details, etc.

Durable efficacy of acupuncture was reported to improve function and alleviate pain for CLBP patients at the individual level [11]. Nonetheless, contradictory results are subsistent [12, 13], that acupuncture efficacy was no better than comforting acupuncture or sham acupuncture [14–16]. Despite negative outcomes of acupuncture for CLBP is not uncommon to discover [13, 14, 17], controversy, and doubting exist in methodological, research environment, unreasonable comparison, multiple acupuncture interventions and study population [18–20]. Firstly, as a complex intervention featured with specific and nonspecific factors [21], acupuncture is not suiting RCT research paradigm that was initiated more appropriately for drug and biomedically-oriented interventions [20]. RCTs is usually limited to strictly selected patients, highly controlled conditions and using a sham intervention so that results are poorly generalizable to real clinical practice [22, 23]. Secondly, very little evidence for CLBP in the special populations such as elder or some subtype of CLBP [24]. Obviously, under real clinical settings, older adults have a higher incidence of CLBP and are likely to suffer more complications [24, 25]. Furthermore, not only the acupuncture style or type, implementation details, treatment duration and frequency of acupuncture can affect outcomes, but also the experience of the operator, the individual conditions that lead to CLBP and the combined treatment too [26, 27]. The lacking of acupuncture experience and perception in the social context may reduce the effectiveness of acupuncture. Most of RCTs was conducted under non-eastern culture, recruited patients lacking acupuncture experience, De Qi perception or cognition were inevitable [28]. Doubtlessly, new research models need to provide more practical details to bridge the gap between rigorous controlled circumstances and real-world practice.

Therefore, we intend to conduct a registry of acupuncture therapy for CLBP through a network platform rooted in the reality of diverse form and operations of acupuncture. This is a prospective, multi-center, registry study. The current status of the study is in recruiting.

The objectives of the study:

- To develop an international registration platform of CLBP for acupuncture therapy;
- To identify the key factors affecting the clinical effectiveness of acupuncture in treating CLBP from the perspective of acupuncture implementation and physicians;
- To analyze population characteristics and subtypes of CLBP suitable for acupuncture.

## Methods

### Study setting

The Registry project of Acupuncture was firstly proposed and inspired at 8th World Conference on Acupuncture WFAS SYDNEY 2013 [29]. According to the principles and framework demonstrated by the Agency for Healthcare Research and Quality [30]. China Association of Acupuncture-Moxibustion (CAAM) had established an Acupuncture Patient Registration Alliance and initiated a network database platform-International Registry Platform of Acupuncture-Moxibustion (IRPAM) in 2017. Application of browser/server architecture based on cloud platform mode and Oracle database development to construct the interactive network database platform.

**Fig. 1:**
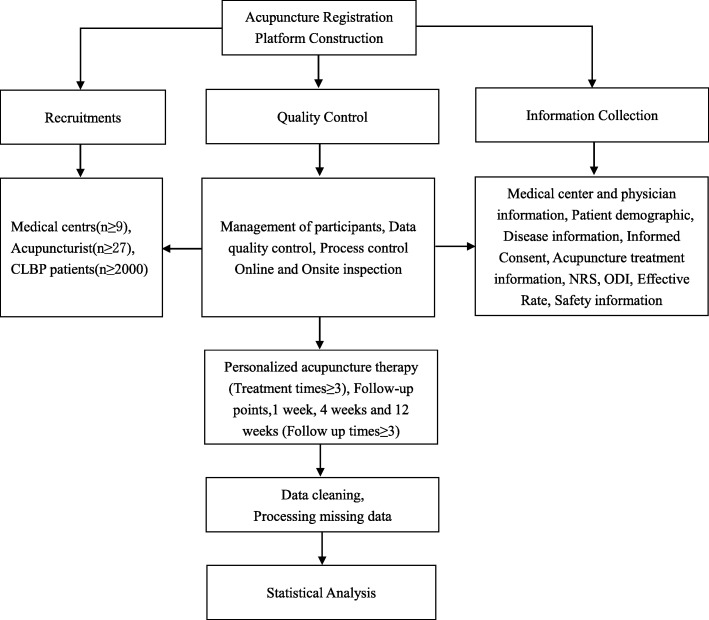
Flow chart of study

The present protocol partially referenced the guideline of the SPIRIT-PRO Extension Checklist [31] and Registries for Evaluating Patient Outcomes: A User’ guide 3th [30]. The technical roadmap is shown in Fig. 1.

### Eligibility criteria

#### Requirements for recruiting physicians

Acupuncture practitioners participating in the study should have the following qualifications:

- Engaged in acupuncture related clinical practice > for 5 years;
- Have a certain amount of outpatient volume of patients with CLBP;
- Prepare individual computer can access to the network;
- Volunteer to participate and sign a memorandum of understanding.

### Patient source

Patients with CLBP were enrolled continuously to reduce selection bias. All recruited patients will be registered dynamically by the acupuncture physicians. The diagnostic criteria, inclusion criteria and exclusion criteria as follows.

### Diagnostic criteria

Pain or muscle tension in the area below the chest and above the buttocks, with or without pain in the lower extremities, and the pain lasts for more than 12 weeks [32].

### Inclusion criteria

- Chronic low back pain or acute onset of chronic low back pain were observed as the main objects to meet the diagnostic criteria for chronic low back pain.
- 16 years≤Age ≤ 80 years;
- The main treatment method is acupuncture.
- Voluntarily participate in the patient registration study and sign informed consent.

### Exclusion criteria

- Patients with severe systemic diseases, such as primary diseases of cardiovascular, liver, kidney and hematopoietic system, and severe mental disorders;
- Patients with backache caused by tumor, tuberculosis, cyst and other reasons.

### Outcome measures

#### Primary outcomes

Considering the convenience of real-world measurements, self-reported disability and pain preferable with CLBP. Therefore, Numerical Rating scale (NRS) [33] and Oswestry Disability Index (ODI) [34] were selected as primary outcomes. These two indexes have been confirmed good validity and reliability in the aspects of back-related pain and disability [35, 36].

### Numerical rating scale (NRS)

Patients were asked to use the numbers 0–10 to quantify pain, with ‘0’ symbolize no pain and ‘10’ symbolize most pain. Higher the number, heavier the pain.

### Oswestry disability index (ODI)

The ODI instrument contains 1 item on pain and 9 items on the activities of daily living (daily activities, lift weights, walk, sit, stand, sleep, and social activities, travel and sex). Each entry from 0 to 5 points to calculate, the cumulative score will be added 2 times and the total score range of 0–100 points. Higher the score, lower the back function.

#### Effective rate

Minimal clinically important difference (MCID) is an index to identify effective group, that is the NRS score decreased by 2 points or ODI score cut down 10 points [36]. The effective number divides the total number of people included in the analysis is the effective rate.

### Secondary indicator

#### Adverse event

Abnormal conditions in the observation period, such as needle dizziness, broken needles, bleeding and hematoma, will be recorded. The occurrence time, cessation time duration, correlation with acupuncture, the measures taken and outcomes of adverse events will be recorded too. Serious adverse events during the observation will be reported promptly.

### Participant timeline

As a dynamic registry design, we plan to stick to the registry for at least 2 to 5 years. The tolerance range of time is 3 days around the follow-up point. If the patient does not return after the last visit, follow-up is done by phone within 1 week. At least ensure follow-up at these three time points,1 week, 4 weeks and 12 weeks. Contents of follow-up: whether there is a recurrence, whether there is back pain related surgery, whether there is any other treatment.

### Sample size

Currently, there is no valid sample size calculation method for registry studies. According to experience, the principle of sample size estimation in multivariate analysis of variance is 5–10 times the number of variables. Our preliminary research variables are no more than 20, so the sample size of the study is 1000–2000, taking the maximum value, our sample size is set at not less than 2000. To ensure better data representation and reflect the diversity of acupuncture operations, we will recruit no less than 9 centers, at least 3 acupuncturists in each center, that is, at least 27 acupuncturists.

### Recruitment

Through academic conferences of the World Federation of Acupuncture and Moxibustion Societies (WFAS) and Chinese Association of Acupuncture-moxibustion (CAAM), we will attract more physicians. In the recruited centers, recruitment announcements will be launched to help patients know more about the program. Moreover, recruiting more clinicians to register on the platform is an important way that can be conducted on the website (www.amreg.org).

### Data elements and collection methods

All information will be collected through a specific network platform for acupuncture therapy and put on record in a paper version (http://www.amreg.org/). The main data elements include information of medical centers and acupuncturists, characteristics of patients with CLBP, acupuncture operation details, outcomes and safety information. Informed consent requires scanning and uploading. The names of diseases related to chronic back pain are identified by International Classification of Diseases (ICD-10,2010 Version) [37]; TCM syndromes are confirmed by Classification and codes of diseases and *ZHENG* of traditional Chinese medicine (GB/T 15657–1995) [38]. The acupuncture point vocabulary adopts the WHO Standard Acupuncture Point Locations in the Western Pacific Region (A WPRO Publication, 2008) [39]. All data elements will be collected using the established terminology specifications, data dictionaries.

Most of the data use structured information entry, allowing free text entry to a certain extent. Platform support Pinyin code retrieval and input and fuzzy matching input to ensure the standardization of registration information and facilitate the statistical analysis of data. Under the premise of ensuring the necessary information, entries should be as little as possible to minimize the burden on clinicians. Specific entries and variables are available in Table 1.

**Table 1:** Metadata of main items and variables

Major data sources	Main items	Property	Main variables and labels
Medical Centre	Institutional background	Indispensable	Medical Organizations, Location and hospital attributes, Medical institution level
	Registration of contribution	Indispensable	Number of recruited practitioners, Number of enrolled patients
Physicians	Demographic Information	Indispensable	Name, Age, Gender, Place of residence, Contact information, Educational background
	Practitioner background	Indispensable	Medical Organizations, Years of employment, Professional qualification (senior or intermediate professional title)
Patients	Demographic Information	Indispensable	Name, Age, Gender, Nationality, ID number, Residence, Occupation, Marriage status. Educational Qualifications, Contact information
	CLBP Information	Indispensable	Visit date, Chief complaint, Course of disease, Location of pain, Characteristics of pain, causes of pain aggravation, Medical history, Causes of pain relief, Back function, Concomitant symptoms, History of acupuncture and Reception, auxiliary examination
	Diagnosis information	Indispensable	Disease diagnosis, Syndrome differentiation of TCM etiology and meridians, Complicating disease
	Lumbar imaging	Optional	X ray, CT examination, Magnetic Resonance Imaging
	Informed Consent	Indispensable	NA
Acupuncture treatment process	Acupuncture rationale	Indispensable	Acupuncture date, Acupuncture style, Acupoint selection principle, Operation of acupoints
	Acupuncture details	Indispensable	Response elicited, Needle retention time, Needle stimulation, Needle type (gauge, length), Adjunctive therapy, Needle parameter, Co-intervention
	Acupuncture regimen	Indispensable	Number of treatment session, Frequency of treatment
	Combined therapy	Indispensable	Pharmacotherapy, Non-pharmaceutical therapy, Other acupuncture type
Outcomes	Effectiveness evaluation	Indispensable	NRS, ODI, Effective Rate
	Safety evaluation	Indispensable	Adverse events
	Follow up	Indispensable	Disease status (Recurrence, Lower back pain related surgery, Seeking other treatments)

*CT* Computed tomography, *NA* Not applicable, *NRS* Numeric rating scale, *ODI* Oswestry disability index, *TCM* Traditional Chinese Medicine

Considering the characteristics and clinical practice of acupuncture therapies, we referenced Standards for Reporting Interventions in Clinical Trials of Acupuncture (STRICTA) [27] to building data dictionaries and frameworks. Structured, standardized, and logical metadata will be accumulated to reflect the real clinical process of acupuncture treatment for CLBP. Baseline demographic data, CLBP characteristics, patient-centered outcomes of the effectiveness and safety will be truthfully recorded. Specific data element entry and minimum dataset in the register can be found in Table 2. Acupuncture treatment and combined treatment should be recorded at each visit. Changes in symptoms, ODI, and NRS scores were recorded before and after treatment for effectiveness evaluation.

**Table 2:** Timetable for data collection

Data elements	Therapeutic Session					Follow-up Period			
	Minimum treatment^a^			…	Last Visit	Minimum follow-up^b^			…
	First Visit	Second Visit	Thirdly Visit			Week 1	Week 12	Week 24	
Demographic information	✓								
CLBP information	✓	✓	✓	✓	✓				
Diagnosis	✓								
Lumbar imaging	✓								
Informed Consent	✓								
Acupuncture rationale	✓								
Acupuncture details	✓	✓	✓	✓	✓				
Acupuncture regimen	✓								
Combined therapy	✓	✓	✓	✓	✓				
Numerical Rating scale (NRS)	✓				✓	✓	✓	✓	
Oswestry Disability Index (ODI)	✓				✓	✓	✓	✓	
Adverse event	✓	✓	✓	✓	✓	✓	✓	✓	
Disease status						✓	✓	✓	✓

^a^The number of acupuncture treatments shall be no less than three times, ^b^No less than three follow-up visits

### Statistical methods

#### Missing data

Although we have internal quality control methods to ensure data integrity, follow-up nonresponse shall be an inescapable challenge in a prospective observation study. Fortunately, we will recruit experienced physicians with stable patient population (at least 5 years of acupuncture experience or middle and senior titles), so the bias for lack of follow-up could be controlled partially. Real-time monitoring of the electronic network platform shall control missing data less than 20%. According to the classical rules of missing classification, follow-up nonresponse belongs to missing not at random (MNAR) [40]. Multiple imputation invented by Rubin shall be adopted to process missing data [41].

#### Analytic approach

Data with poor compliance, non-compliance with the study program or missing important information are not included in the analysis. Data processing and statistical analysis will use SPSS version 24 (IBM Corp., Armonk, NY, USA) and SAS Version 9.4 (SAS Institute Inc., NC 27513–2414, USA).

We will select 1-week,4-week,12-week, assessments respectively at evaluation time point on the ODI and NRS index. Firstly, baseline characteristics comparability needs to be assessed between the propensity-matched groups. Model adjustment suggested by Rosenbaum and Rubin will be adopted to conquer residual covariance imbalances [42]. Secondly, multivariate linear regression models will be employed to adjust the propensity score and baseline characteristics. Once matched, change in ODI and NRS between-group at different observation points will be calculated by paired t-test. Thirdly, logistic regression models will be used to evaluate the magnitude of impacts contributed by baseline factors on acupuncture effectiveness for CLBP patients. Descriptive analysis will be used for variables such as the recruited research center,s basic characteristics of doctors and patients, initial diagnosis and intervention details. Quantitative data (Height, Weight, NRS, ODI, etc.) will be described by mean, standard deviation, median, maximum and minimum values. The enumeration data (Acupuncture style, Acupoint selection principle, Operation of acupoints, Effective rate etc.) and ranked data (Educational background, Physician title) will be described by numbers, percentages, rate and constituent ratio. If necessary, parametric methods or alternate nonparametric methods will be selected appropriately.

We will adopt the propensity score matching of patients at 1:1 to imitate an RCT. Baseline confounder will be equilibrated and matched on variables such as demographic data, medical history, duration, intensity, back function. The comparative analysis will be conducted after the baseline unevenness adjustment. Propensity score will match comparison group, such as Traditional Chinese acupuncture style vs Microacupuncture style (Scalp acupuncture, Ear acupuncture, Wrist and ankle acupuncture, Eye acupuncture, Umbilical acupuncture, Abdomen acupuncture), Local acupoint selection vs Non-local acupoint selection and Single Acupuncture vs Combined therapy etc.

Hierarchical models based on the pain grading, medical center, age group, gender, TCM syndromes and classification of diseases will be taken into account to explore the important factors affecting the effectiveness of acupuncture for CLBP [43–45]. Conditional logistic regression models adjusting by different variables to identify predictors of long-term effectiveness presented by ODI and NRS or recurrence at the12-week follow-up.

### Quality management

#### Management of participants

Before acupuncture physicians are confirmed to be enrolled, they need to apply for an individual account on the registration platform. Only after the subject research management unit has reviewed their information and approved can they join the project. All organizational personnel, platform developers and acupuncturists are required to participate in standardized training and must acquaint with an investigator’s manual.

#### Data monitoring

Considering the differences in the filling of registry information between different doctors, standardizing and integrating of diagnosis is the main concern. Data Monitoring will conduct online or on-site quality inspection on the registered information every month, and timely feedback the problems. The concern includes the accuracy, integrity, authenticity and timeliness of the registry. Once problems confirmed, verification or traceability will be conducted timely. Meanwhile, problems existing in the research process will be posted and timely solved via network or telephone. For registered contents that are not clearly expressed, such as contradictory information, important data missing. Once verified, online modification will be carried out and recorded for later reference.

#### Data quality management

During the data collection process, the platform can proceed with template management, according to the time verification, integrity, validity, and logical verification. Furthermore, the ID number is the unique identification code of the patient on the platform to avoid duplicate entry. The corresponding prompt interface will appear to reduce the wrong data and missing data to facilitate data quality.

Preliminary manual verification of data will be conducted quarterly and timely feedback to the research team. A training conference on registry implementation and problem summary will be conducted every year to gradually summarize the experience. Therefore, the accuracy, fidelity and timeliness of data quality will be improved gradually. A data quality and safety oversight committee will be established to conduct regular assessments of data quality and safety, as well as of acupuncturists. Implement management of procedures, policies, procedures to ensure the quality of data within the registry and control the following errors.

Entry errors: such errors can be avoided through prior data quality checks (such as value range and validity checks), prompt the data that does not conform to the regulation when the system input and strict data cleaning; Biased errors: mainly by checking the continuity of data collection, checking the registered information with the ID and conducting on-site inspections randomly.

#### Data cleaning and screening

Cleaning Principles: keeping the original data permanently to guarantee traceability, keeping the original case information unchanged, data were transformed, split, extracted and merged, etc. Specifically, the standardization of the special acupoints, such as ‘Huatuo jiaji’ will be standardized as ‘Waist jiaji point’, ‘Ashi’ was standardized as ‘Ashi point’.

#### Authority management

To ensure the security and privacy of study data, different access rights will be set for users in the platform. Permissions access mainly contains the operation level and data level, according to different users demands and role in the program. According to the permit classifications, platform will accomplish the access control of operational level and data level. Administrators can check, lock, initialize passwords, set permissions, templates and other basic functions of doctors.

#### Data security and patient privacy protection

Data access shall be strictly controlled, and qualification examination shall be carried out for those who log on to the Registry platform. Only researchers and quality inspectors participating in the program have access to the personal medical records of subjects. Clinical registered doctors are only allowed to access their own patients. Sensitive information of patients such as name, ID number and contact information is processed by encryption technology. In data transmission, SSL (Secure Sockets Layer) is used for encryption, and switched virtual network system is used for client access. Encrypted storage of sensitive information of registered doctors and patients, the data should be backed up regularly every week. The completed case information should be archived and stored locally, limiting access rights. The access of storage server also adopts strict authorization mechanism. Each user occupies a unique account in the system and grants different permissions to different researchers.

### Dissemination policy

The research data will be published in the form of a paper after the summary analysis. Different research centers can use the data collected by their own centers to publish, but must pass the review of the research team.

## Discussion

Some registry studies or programs related to low back pain have been established worldwide, such as Quebec registration [46], BOLD registry [25], PRECISION registry [47], etc. Nevertheless, no registration specifically for acupuncture therapy details of CLBP. Unlike previous studies were designed to explain the efficacy of acupuncture. We plan to explore the characteristics of acupuncture implementation and conditions related to more acupuncture benefit for CLBP. Depending on standardized, structured and high-fidelity data collection, we will have a more comprehensive understanding of acupuncture treatment. Through comparisons of different acupuncture stimulation parameters, we will explore the best acupuncture treatment programs for different states and subtypes of CLBP.

Considering maneuverability and convenience of registration implementation, we have carefully considered research design. Logic verification of structured data acquisition and variable input ensures the correctness and completeness of the data. The relatively short follow-up period and convenient data entry process will reduce the data entry burden of clinical acupuncturists and ensure smooth operation. What’s more, the STRICTA rules and TCM syndrome standard entries are adopted to ensure data integrity and standardization. NRS and ODI scale can be completed in 5 min, the respondent burden is low [35]. Beyond that, ODI and NRS have been widely used in researches [13, 36, 48, 49] for CLBP because of robust psychometric information [35, 50]. We originally intended to cover patients of all ages in the patient group, but considering the actual situation of acupuncture recruitment and the response-ability of the scale, we excluded patients older than 80 years and younger than 16 years. Although many studies have used psychosocial-related scales like Perceived self-efficacy [51], Fear-avoidance beliefs [52], Depression Anxiety Stress Scale [53, 54] as indicators or influencing factors for the evaluation of chronic low back pain [43]. We do not adopt these indicators based on some practical considerations. A study has shown that scales supporting acupuncture practice should be considered more often by the acupuncturists to evaluate acupuncture in CLBP [45]. Tedious items and questionnaires of these scales may increase patient resistance and interfere with our judgment on the effectiveness of treatment. Besides, we believe that the psychological factors themselves are embedded in the communication between the acupuncturist and the patients during the acupuncture process. Therefore, the assessment based on the details of the implementation itself also includes the evaluation of psychological related factors. Most importantly, the psychological state and the pain experience itself are difficult to peel off [51, 55]. Thence, psychological scales are not the preferred indicator for this research study.

Evidence from registry study will be an important supplement to the RCTs. Through the acupuncture registry study for CLBP, we will at least benefit in the following aspects. Firstly, gaining more practical and reliable evidence for acupoint selections and guideline modifications. Secondly, to explore potential confounding factors of acupuncture implementation and provide ideas for high-quality RCTs in the future. Thirdly, through the accumulation of potential acupuncture adverse reactions and excavation of big data, it will be helpful to scientifically choose an individual, safe, and efficient acupuncture style. Characteristics of acupuncture ineffective cases will be identified, avoiding waste of medical resources or delay the patient’s condition. Fourthly, the registration organization will help recruit excellent professional acupuncture teams for the treatment of CLBP, providing a better and standardized acupuncture program for the treatment of CLBP. Therefore, our evidence from a large sample, real-world settings and consistent with the characteristics of acupuncture will be indispensable.

### Strengths of study

As far as we know, there are very few registries for acupuncture, which is our biggest innovation. We can obtain responsibly data that relatively high-quality and characterized by acupuncture through structured, standardized, and electronic data collection. Furthermore, prospective data come from real-world will be more convincing than retrospective data. Our registry projects will provide an applicable paradigm for research of complementary and alternative medicine.

### Limitations of study

In the initial stage, center selection bias maybe be inevitably affected by real recruitment results, we will promote geographical representation by expanding recruitment. Lacking the pre-defined comparable group will be the inevitable limitation, as well as selection bias. Although we will adopt the method of propensity score matching to implement post hoc randomization and conduct stratification analysis, hidden, unknown confounding factors will still exist. Perhaps, in future research, we can embed pragmatic randomized controlled trials, pRCTs with long-term follow-up into the platform as a sub-project, to obtain more reliable and abundant evidence [23, 56].

## Conclusion

This study will identify key factors that influence the effectiveness of acupuncture and explore effective acupuncture interventions with different characteristics of low back pain. Furthermore, reliable acupuncture evidence from the registry will be an important supplement to the future RCTs for the treatment of CLBP.

## Data Availability

Data sharing is through the network platform (http://www.amreg.org/), different access rights will be set for users in the platform.

## Abbreviations

CAAM: Chinese Association of Acupuncture-moxibustion
CLBP: Chronic low back pain
HIS: Hospital information system
ID: Identification
IRPAM: International Registry Platform of Acupuncture-Moxibustion
MCID: Minimal clinically important difference
MNAR: Missing not at random
NRS: Numeric rating scale
ODI: Oswestry Disability Index
RCTs: Random controlled trials
SSL: Secure Sockets Layer
STRICTA: Standards for Reporting Interventions in Clinical Trials of Acupuncture
TCM: Traditional Chinese Medicine
WFAS: World Federation of Acupuncture-Moxibustion Societies
WPRO: Western Pacific Region Office
